# Genome Sequence of *Streptomyces exfoliatus* DSMZ 41693, a Source of Poly(3-Hydroxyalkanoate)-Degrading Enzymes

**DOI:** 10.1128/genomeA.01272-13

**Published:** 2014-02-06

**Authors:** Virginia Martínez, Daniel Hormigo, Carlos del Cerro, Patricia Gómez de Santos, Javier García-Hidalgo, Miguel Arroyo, Auxiliadora Prieto, José Luis García, Isabel de la Mata

**Affiliations:** a Department of Biochemistry and Molecular Biology, Facultad de Biología, Universidad Complutense de Madrid, Madrid, Spain; b Department of Environmental Biology, Centro de Investigaciones Biológicas, Consejo Superior de Investigaciones Científicas, Madrid, Spain

## Abstract

Here we report the draft genome sequence of *Streptomyces exfoliatus* DSMZ 41693, which includes a gene encoding a poly(3-hydroxyoctanoate) depolymerase, an enzyme which can be used for the industrial synthesis of chiral (*R*)-3-hydroxyalkanoic acids. In addition, the genome carries numerous genes involved in the biosynthesis of secondary metabolites, including polyketides and terpenes.

## GENOME ANNOUNCEMENT

Streptomycetes, widely distributed filamentous aerobic Gram-positive bacteria, have complex life cycles that include programmed cell death (PCD) and sporulation (1). They produce two-thirds of antibiotics (2, 3) and represent an important source of novel bioactive compounds as well as of many enzymes for food manufacturing (4) and industrial (5, 6) and environmental applications (7, 8). Several *Streptomyces* genomes have been completely sequenced in recent years and numerous genome sequencing projects with different *Streptomyces* species are ongoing. Streptomycetes have linear chromosomes (approximately 8 to 12 Mb) with high G+C content levels (3). More than 20 diverse secondary metabolic gene clusters in their genomes have been described to date (3, 9).

Among streptomycete strains, *Streptomyces exfoliatus* was identified as a poly(3-hydroxyalkanoate) (PHA)-degrading strain (10). This bacterium is distinguished from other PHA-degrading bacteria by its ability to degrade both *scl*-PHA, such as poly(3-hydroxybutyrate) (PHB), and *mcl*-PHA, such as poly(3-hydroxyoctanoate) (PHO) (10). A PHA depolymerase-encoding gene of *S. exfoliatus* (*phaZ*_Sex_) has been cloned and expressed in *Rhodococcus* sp. strain T104 and its product was functionally characterized (5). This depolymerase was specific for PHB and did not hydrolyze PHO, indicating the presence of at least one additional gene in *S. exfoliatus* which encodes a PHO depolymerase.

Here we present the draft genome sequence of *S. exfoliatus* DSMZ 41693, which was obtained from a shotgun library constructed and sequenced using a Titanium kit in a 454 GS-FLX instrument (Roche Diagnostics, Branford, CT) at Lifesequencing S.L. A total of 4 × 10^6^ reads with a mean size of 531 bp were assembled by Newbler 2.5.3 software, generating 367 large contigs and providing 26.1-fold coverage. The open reading frames (ORFs) and RNA genes were predicted by the RAST server (11).

The draft genome of *S. exfoliatus* DSMZ 41693 comprises 8.8 Mbp, with a 71.7% G+C content. There are 8,110 predicted ORFs in the genome, among which 4,996 ORFs have putative assigned functions, as well as 73 RNA-encoding genes. *S. exfoliatus* contains the Embden-Meyerhof pathway and tricarboxylic acid (TCA) and glyoxylate cycles.

Furthermore, the genome possesses numerous genes of PHA metabolism, including 48 genes of PHB metabolism and a gene coding a putative PHO depolymerase, presenting *S. exfoliatus* as a possible microbial source for obtaining a novel enzyme with potential applications in the production of (*R*)-3-hydroxyalkanoic acids as well as in degradation of bioplastics or biomaterials. So far, 10 diverse secondary metabolism genes have been identified, including those for the synthesis of polyketides and terpenes; these genes are located on the *S. exfoliatus* genome in various gene clusters, which exhibit high genomic synteny to those of several *Streptomyces* strains. The *S. exfoliatus* DSMZ 41693 genome also contains genes encoding cellulases, amylases, xylanases, and chitinases, as well as genes encoding proteases, lipases, and esterases. Genes involved in the resistance to heavy metals were also identified, indicating the potential application of this strain in biomass conversion and environmental bioremediation.

### Nucleotide sequence accession number.

The genome sequence determined in this whole-genome shotgun project has been deposited at Genbank under accession number AZSS00000000.
